# Older Age Does Not Have an Impact on Postoperative Complication Risk Following Transurethral Resection of the Bladder Tumor

**DOI:** 10.3390/curroncol33080457

**Published:** 2026-07-30

**Authors:** Minami Une, Shinya Yamamoto, Honoka Fuse, Yusuke Yoneoka, Kenjiro Noda

**Affiliations:** Department of Urology, Nishitokyo Chuo General Hospital, Tokyo 188-0014, Japan; s_yamamoto4@tmg.or.jp (S.Y.); h_fuse@tmg.or.jp (H.F.); y_yoneoka@tmg.or.jp (Y.Y.); k_noda@tmg.or.jp (K.N.)

**Keywords:** age, bladder cancer, older patients, postoperative complications, risk factors, transurethral resection of bladder tumor (TURBT)

## Abstract

Background/Objectives: The incidence of bladder cancer has been increasing within the aging population. Transurethral resection of bladder tumor (TURBT) is the first-line treatment for bladder cancer, serving both diagnostic and therapeutic purposes. In this study, we aimed to investigate the incidence and predictors of postoperative complications following TURBT in a general hospital with a high proportion of older patients. Methods: We analyzed 141 Japanese patients retrospectively who had undergone TURBT for clinically diagnosed bladder cancer between January 2018 and December 2023. Postoperative complications occurring within 30 days were reviewed from medical records, and risk factors were evaluated. Results: Postoperative complications were observed in 49 patients (34.8%), with 28 patients (20%) classified as Clavien–Dindo grade I and 21 (15%) as grade II or higher. In univariable analysis, factors significantly associated with postoperative complications included older age (odds ratio [OR]: 1.04; *p* = 0.027), lower platelet count (OR: 0.91; *p* = 0.009), positive urine cytology (class III or higher) (OR: 2.69; *p* = 0.007), pathological T2 or higher stage (OR: 5.79; *p* = 0.013), larger tumor size (OR: 1.05; *p* = 0.005), and longer operative time (OR: 1.02; *p* = 0.009). In multivariable analysis, independent risk factors for postoperative complications were lower platelet count (OR: 0.89; *p* < 0.01), pathological T2 or higher (OR: 5.51; *p* = 0.03), and larger tumor size (OR: 1.05; *p* < 0.01). Notably, older age was not an independent risk factor. Conclusions: TURBT appears feasible in older patients when performed by experienced surgeons. Age alone should not be considered a limiting factor for surgical eligibility.

## Simple Summary

Transurethral resection of bladder tumor (TURBT) is the standard initial treatment for bladder cancer, particularly in aging populations. We analyzed 141 Japanese patients retrospectively who had undergone TURBT to identify the frequency and risk factors for postoperative complications. Complications within 30 days occurred in 34.8% of patients, with most being mild. Univariable analysis identified older age, lower platelet count, positive urine cytology, larger tumor size, longer operative time, and pathological stage T2 or higher as factors associated with postoperative complications. However, multivariable analysis showed that only lower platelet count, pathological stage T2 or higher, and larger tumor size were independent risk factors. Importantly, age itself was not independently associated with postoperative complications. These findings suggest that older age alone should not preclude TURBT when appropriate perioperative management is provided, and that advanced tumor characteristics and hematologic status may be more important considerations than age alone when evaluating surgical risk.

## 1. Introduction

Bladder cancer ranks as the ninth most prevalent cancer in the world, with 613,791 patients newly diagnosed each year, and 220,349 deaths caused by the disease [1]. In general, many cancers, including bladder cancer, are considered a disease of the older population [2,3]. Japan’s aging population is advancing; therefore, bladder cancer is also likely to increase further in the future [2,4]. Consequently, the number of transurethral resection of bladder tumor (TURBT) procedures performed in older adults has also shown an increasing trend. TURBT remains the cornerstone of bladder cancer management. It provides pathological confirmation of the diagnosis and enables accurate assessment of tumor grade and stage [5]. In addition, TURBT is essential for determining subsequent treatment strategies, including intravesical therapy, repeat TURBT, radical cystectomy, or systemic treatment [6]. Besides its diagnostic role, TURBT can provide effective local tumor control and symptom relief, particularly in patients presenting with hematuria or lower urinary tract symptoms [5]. Because non-muscle-invasive bladder cancer is characterized by a high risk of recurrence and progression, most patients require long-term cystoscopic surveillance after TURBT. Furthermore, repeat TURBT may be necessary in selected patients with recurrent or high-risk disease. Thus, TURBT plays many important roles. Therefore, understanding the safety profile of TURBT is important not only for initial treatment but also for long-term disease management [7]. Thus, assessing postoperative outcomes after TURBT is clinically important, particularly in elderly patients who often require individualized treatment planning.

Advanced age is generally considered a risk factor for adverse postoperative outcomes [8,9]. Age-related declines in cardiovascular and pulmonary function may increase vulnerability to perioperative stress [9]. As a result, treatment decisions for elderly bladder cancer patients are often more complex than those for younger patients, requiring careful consideration of both oncological benefit and treatment-related risk. However, chronological age does not necessarily reflect biological age or functional status. Frailty has emerged as an important concept in the management of elderly cancer patients. Frailty is characterized by a decline in physiological reserve and an increased vulnerability to external stressors, resulting in a reduced ability to recover from surgical interventions. Several studies have demonstrated that frailty is associated with a higher risk of postoperative complications, prolonged hospitalization, functional decline, and mortality across various surgical specialties [10,11]. In contrast, some older individuals maintain good physical function and independence despite advanced chronological age, highlighting the heterogeneity of the elderly population. Comprehensive geriatric assessment (CGA) has been increasingly recommended as a tool for evaluating elderly patients before cancer treatment. CGA assesses multiple domains, including functional status, comorbidities, cognition, nutrition, psychological condition, and social support. Previous studies have suggested that CGA may improve risk stratification and help identify patients who are suitable candidates for surgical treatment [12,13]. These findings suggest that treatment decisions should not be based solely on chronological age but should incorporate a broader evaluation of the patient’s overall health status and physiological reserve. As life expectancy continues to increase worldwide, the number of elderly patients undergoing surgical treatment for bladder cancer is expected to rise substantially. Establishing evidence-based criteria for surgical decision-making in this population has become an increasingly important clinical issue. Clarifying whether age itself contributes to postoperative complications may help avoid unnecessary undertreatment of elderly patients who could potentially benefit from TURBT. Recent studies have suggested that factors such as frailty, nutritional status, and comorbidities may have a greater impact on postoperative outcomes than age itself [8,14]. Evidence regarding the association between age and complications following TURBT remains limited and inconsistent. Some studies have reported a higher incidence of perioperative morbidity among elderly patients [15], whereas others failed to identify age as an independent predictor of complications [16]. Whether advanced age alone should influence surgical decision-making for TURBT remains unclear. To address this issue, we investigated whether age was a risk factor for complications following TURBT.

## 2. Materials and Methods

Between January 2018 and December 2023, a total of 141 Japanese patients with clinically diagnosed bladder cancer on cystoscopy who had undergone TURBT were included in this study. The inclusion criteria were patients aged ≥20 years who underwent TURBT for bladder tumors diagnosed by cystoscopy and/or imaging studies. Patients with both initial and recurrent bladder tumors were included in the analysis. Recurrent tumors were defined as bladder tumors detected after previous TURBT or intravesical treatment for bladder cancer. The exclusion criteria included patients with incomplete clinical data, non-urothelial carcinoma confirmed on pathological examination, concomitant upper tract urothelial carcinoma, and patients who underwent emergency TURBT because of uncontrolled gross hematuria or urinary obstruction. All patients underwent routine preoperative evaluations including blood examinations, chest radiography, electrocardiography, echocardiography, and pulmonary function testing when clinically indicated. Anticoagulant and antiplatelet medications were managed according to institutional protocols and perioperative bleeding risk. All TURBTs were performed by one experienced urological surgeon. All operations were performed using a bipolar electrosurgical system and a 24 Fr resectoscope (Olympus Corporation). The coagulation and cutting settings were 80 and 120 W, respectively. After all visible tumors had been resected, the margin was excised with approximately a 5-mm clearance from the lesion. After TURBT, an 18 Fr catheter was placed, and was usually removed on postoperative day 3. Gross hematuria is defined as urine appearing bright red to dark reddish-brown by the naked eye [17]. Data at TURBT on age, sex, Eastern Cooperative Oncology Group Performance Status (ECOG PS), body mass index (BMI), preoperative cytology, tumor size, pathological findings, Charlson comorbidity index (CCI), solitary or not, times of TURBT, operation time, white blood cell (WBC), hemoglobin, platelet count, creatinine, albumin, alkaline phosphatase (ALP), corrected calcium, C-reactive protein (CRP), sodium (Na), potassium (K), chloride (Cl), and calcium (Ca) were collated from the medical records. For patients with multiple tumors, tumor location was classified according to the dominant tumor. Pathological diagnoses were made by board-certified pathologists at our institution. Preoperative urine cytology was evaluated according to the Papanicolaou five-class classification system. For statistical analyses, Classes I–II were classified as negative, whereas Classes III–V were classified as positive. Pathological staging was performed according to the Tumor–Node–Metastasis (TNM) classification system, and tumor grading was assessed according to the World Health Organization (WHO) classification. All pathological specimens were independently evaluated by experienced genitourinary pathologists at our institution. In cases of diagnostic uncertainty, pathological findings were reviewed and discussed to achieve consensus. BMI was calculated using the formula: BMI (kg/m^2^) = ([weight]/[height]^2^). The Charlson Comorbidity Index (CCI) was calculated by reviewing patients’ medical records to identify pre-existing comorbidities according to the original Charlson Comorbidity Index. Age was not included in the CCI score because age was analyzed as an independent variable [18]. Complications occurring within 30 days after TURBT were retrospectively assessed through review of inpatient records, outpatient follow-up records, and readmission data. Both medical and surgical complications were included in the analysis. Postoperative complications were identified based on physician documentation and objective clinical findings. All complications were classified according to Clavien–Dindo classification grading [19,20]. Consistent with the published application of the Clavien–Dindo classification for TURBT [21], transient gross hematuria managed conservatively without blood transfusion or surgical intervention was classified as a Grade I complication. Logistic regression analysis was used to identify factors associated with postoperative complications. All statistical analyses were conducted using EZR (Saitama Medical Center, Jichi Medical University, Saitama, Japan), which is a graphical user interface for R (R Foundation for Statistical Computing, Vienna, Austria). Continuous variables were expressed as medians and interquartile ranges, whereas categorical variables were expressed as frequencies and percentages. Comparisons between groups were performed using the Mann–Whitney U test for continuous variables and Fisher’s exact test for categorical variables, as appropriate. A *p*-value < 0.05 was considered statistically significant. Variables with *p* < 0.10 in the univariable analysis were entered into the multivariable model. Because age was the primary variable of interest in this study, it was forced into the multivariable model regardless of the variable selection procedure. Multicollinearity among the explanatory variables was assessed using the variance inflation factor (VIF), and no significant multicollinearity was observed. Odds ratios (ORs) and 95% confidence intervals (CIs) were calculated.

Generative artificial intelligence (ChatGPT, GPT-5.5, OpenAI) was used solely to assist with English language editing and improve the clarity and readability of the manuscript, including revisions made during the peer-review process. No generative AI tools were used for the study design, data collection, data analysis, interpretation of the results, or formulation of the scientific conclusions. All AI-assisted text was carefully reviewed and edited by the authors, who take full responsibility for the accuracy and integrity of the manuscript.

## 3. Results

Table 1 shows the demographic data of all 141 patients. Of the 141 patients, 113 were male and 28 were female. The median age was 77 years. Pathological stage no malignancy, Ta, T1, pure carcinoma in situ (CIS), and T2 or higher were 17, 85, 23, 7, and 9, respectively; 97 patients (69%) had high-grade cancer. The median Charlson Comorbidity Index was 5, and most patients had a good performance status, with 126 patients (89%) classified as ECOG PS 0. The median tumor size was 15 mm and the median operative time was 50 min.

Table 2 shows all postoperative complications. The postoperative complications occurred in 49 patients (34.8%) and, of those, 28 and 21 patients (20% and 15%) were classified as Clavien–Dindo grade I and grade II or higher, respectively. Of all complications, gross hematuria was the most common complication, followed by urinary retention and bladder perforation. Severe complications (Clavien–Dindo grade III or higher) were observed in eight patients.

Table 3 shows the association of variables with complications in univariable and multivariable analyses. In the univariable analysis, the following variables were significantly associated with complications: age (OR [odds ratio]: 1.04; *p* = 0.027), platelet (OR: 0.91; *p* = 0.009), cytology class III or higher or higher (OR: 2.69; *p* = 0.007), pathological T2 or higher (OR: 5.79; *p* = 0.013), larger tumor size (OR: 1.05; *p* = 0.005), and longer operation time (OR: 1.02; *p* = 0.009). Variables with *p* < 0.10 in the univariable analysis were entered into the multivariable model. In the multivariable model including age, platelet count, tumor size, pathological stage, urine cytology, and operative time, age was not independently associated with postoperative complications. In contrast, lower platelet counts (OR 0.89, *p* < 0.01), pathological stage T2 or higher (OR 5.51, *p* = 0.03), and larger tumor size (OR 1.05, *p* < 0.01) remained independent risk factors.

## 4. Discussion

We showed that advanced age did not affect the occurrence of postoperative complications following TURBT. In general, as many older cancer patients have chronic diseases, polypharmacy, and decreased function of vital organs, clinicians often anticipate a higher risk of postoperative complications and may hesitate to perform surgery. Several studies have demonstrated that advanced age is associated with increased postoperative morbidity and mortality across a variety of malignancies. Tanaka et al. reported that older cancer patients had significantly higher rates of pulmonary complications, infections, and cardiovascular events after surgery [14]. Similarly, population-based studies have demonstrated that advanced age is associated with increased cancer-related mortality following surgical treatment [22]. There have also been some reports detailing complications following TURBT. Matulewic et al. analyzed the risk factor of complications following TURBT using a multicenter database and reported that only operation time was a significant risk factor [23]. Meanwhile, Pereira et al. also conducted the same analysis using the National Surgical Quality Improvement Program database and reported that an age of more than 80 years indicated a significant borderline increase in perioperative morbidity (*p* = 0.06) [24]. However, evidence regarding the impact of age on outcomes after TURBT remains inconsistent. In our cohort, age was associated with postoperative complications in univariable analysis but lost significance after adjustment for other variables. This finding suggests that factors accompanying aging, rather than chronological age itself, may be more relevant determinants of perioperative risk. Similar observations have been reported by Hollenbeck et al., who identified nutritional and physiological factors, but not age, as independent predictors of postoperative complications following TURBT [25].

One possible explanation for this finding is that TURBT is generally considered a relatively minimally invasive surgical procedure compared with radical surgery. Improvements in perioperative management, anesthetic techniques, and postoperative monitoring may also contribute to reducing surgical risk even in elderly patients. Elderly patients selected for surgery in daily clinical practice are often carefully evaluated before TURBT through comprehensive preoperative assessments, including echocardiography and pulmonary function testing, which may contribute to reducing the incidence of severe postoperative complications.

Lower platelet counts were independently associated with postoperative complications in the present study. Platelets play a central role in hemostasis, and lower platelet counts may increase susceptibility to perioperative bleeding events. Previous studies in general surgical populations have also demonstrated an association between low platelet counts and increased postoperative morbidity [26]. Because platelet count is routinely available before surgery, it may help identify patients who require closer perioperative surveillance. In our cohort, hematuria was the most common postoperative complication. The observed association between lower platelet counts and postoperative complications is biologically plausible, as lower platelet counts may predispose patients to perioperative bleeding after TURBT.

Larger tumors and pathological stage T2 or higher were also identified as independent risk factors for postoperative complications because larger and more invasive tumors often require wider and deeper resections, resulting in greater surgical complexity. Such procedures may increase the risk of postoperative complications, consistent with previous reports [22]. These results suggest that tumor characteristics may have a greater influence on postoperative outcomes than patient age alone.

Interestingly, neither CCI nor ECOG performance status was significantly associated with postoperative complications. Although these variables are commonly used to evaluate the overall health status of cancer patients and have been reported to predict postoperative outcomes in various surgical settings [14], their predictive value may be limited in relatively minimally invasive procedures such as TURBT. Furthermore, CCI primarily reflects the burden of chronic comorbidities and may not fully capture frailty, functional reserve, or physiological resilience, which are increasingly recognized as important determinants of surgical outcomes in older adults [11,14]. Although comorbidity and functional status were assessed using CCI and ECOG Performance Status, validated frailty assessment tools such as the Clinical Frailty Scale and Geriatric-8 were not routinely available in our retrospective cohort.

Gross hematuria was the most frequently observed postoperative complication in our cohort. Because TURBT involves resection of highly vascular bladder tumors and intentional disruption of the bladder mucosa, postoperative bleeding is an expected adverse event. Although most episodes of hematuria were managed conservatively and resolved without invasive treatment, persistent bleeding may lead to urinary retention, clot formation, prolonged catheterization, unplanned outpatient visits, additional interventions, or readmission. The postoperative complication rate in our study appears higher than that reported in some previous studies. This difference may be explained by our comprehensive assessment of all complications occurring within 30 days after surgery, including Clavien–Dindo grade I events. In addition, the high frequency of gross hematuria contributed substantially to the complication rate.

Taken together, our findings indicate that lower platelet count, larger tumor size, and pathological T2 or higher should be considered when assessing the risk of postoperative complications. These factors may contribute to increased bleeding risk through impaired hemostasis or the need for more extensive tumor resection. Accordingly, postoperative monitoring should be particularly careful in patients with these characteristics. Appropriate catheter management and prompt recognition of clot retention or persistent hematuria may help minimize the clinical consequences of postoperative bleeding.

Our findings have important clinical implications for the management of elderly patients with bladder cancer. In clinical practice, some elderly patients may be denied potentially beneficial procedures because of concerns regarding surgical risk. However, avoiding TURBT solely because of chronological age may result in delayed diagnosis, inaccurate pathological staging, and missed opportunities for appropriate treatment selection. Since TURBT remains the cornerstone of bladder cancer management [5,6], treatment decisions should be based on a comprehensive evaluation of the patient’s overall condition, expected therapeutic benefit, and individualized risk profile rather than age alone.

Frailty assessment represents an important area for future investigation. Previous studies have suggested that frailty, nutritional impairment, sarcopenia, cognitive dysfunction, and comorbidity burden may predict adverse postoperative outcomes more accurately than chronological age [11]. Incorporation of validated geriatric assessment tools, such as the Clinical Frailty Scale or Geriatric-8 screening tool, may improve perioperative risk stratification and facilitate individualized treatment planning [27]. Future prospective multicenter studies incorporating frailty assessment and comprehensive geriatric evaluation are warranted to clarify which elderly patients are most likely to benefit from TURBT while maintaining acceptable surgical risk.

There are several limitations to this study. First, this was a retrospective single-center study with a relatively small sample size, which may have introduced selection bias and limited the generalizability of the findings. Because data were collected retrospectively from medical records, some clinical variables may not have been uniformly documented, resulting in potential information bias. Second, all procedures were performed by a single experienced surgeon. Although this reduced variability in surgical technique and perioperative management, the results may not be directly applicable to institutions with different levels of surgical experience. Third, frailty and comprehensive geriatric assessment were not evaluated. Consequently, we were unable to determine whether physiological aging had a greater influence on postoperative complications than chronological age. Future prospective studies incorporating validated frailty assessment tools are warranted. Fourth, complications were assessed retrospectively, and minor complications managed outside our institution may not have been fully captured. Therefore, the true incidence of postoperative complications may have been underestimated. Finally, long-term oncological and functional outcomes were not assessed because the primary objective of this study was to evaluate short-term postoperative complications occurring within 30 days after surgery. Although single-surgeon studies may reduce external validity, they also provide several methodological advantages. Surgical technique, perioperative management, and postoperative follow-up protocols were relatively standardized throughout the study period. This may have reduced procedural variability and enabled a more accurate assessment of patient-related and tumor-related risk factors for postoperative complications. Information regarding perioperative anticoagulant and antiplatelet therapy, anesthesia method, preoperative urinary tract infection, and immediate postoperative intravesical instillation was not available and these potential confounding factors could not be included in the present analyses. Accordingly, caution is required when generalizing our findings to other institutions with different patient populations, surgical expertise, and perioperative management protocols.

Our findings suggest that elderly patients should not be excluded from TURBT solely because of chronological age. Careful perioperative assessment, optimization of modifiable risk factors, and individualized treatment strategies may be more important than age itself in reducing postoperative complications.

## 5. Conclusions

If the patient is given a detailed general condition assessment, then TURBT should be performed by surgeons who are experienced. Even if the patient is older, urologists should positively recommend TURBT for these patients with bladder cancer.

## Figures and Tables

**Table 1:** Baseline characteristics of patients with NMIBC (*n* = 141).

		Total (*n* = 141) ^1^	Complications (*n* = 49)	No Complications (*n* = 92)	*p*
Observation period (months)		33.5 (17.2–54.9)	28.8 (15.9–47.4)	36.7 (20.2–57.4)	0.16
Outcome					0.34
	Dead	12 (9)	6 (50)	6 (50)	
	Alive	129 (91)	43 (33)	86 (67)	
Sex					1.00
	Male	113 (80)	39 (35)	74 (65)	
	Female	28 (20)	10 (36)	18 (64)	
Age (years)		74 (70–83)	78 (72–86)	75 (67–82)	0.03
CCI		5 (4–7)	5 (5–7)	5 (4–7)	0.16
ECOG PS					0.29
	0	126 (89)	41 (33)	85 (67)	
	1	8 (6)	5 (63)	3 (37)	
	2	1 (1)	0 (0)	1 (100)	
	≥3	6 (4)	3 (50)	3 (50)	
BMI (kg/m^2^)		23.5 (21.4–25.3)	23.5 (21.6–26.3)	23.6 (21.4–25.2)	0.82
LDH (U/L)		183 (162–212)	183 (164–209)	185 (162–219)	0.69
ALP (U/L)		116 (75.8–219)	113 (71–201)	130 (77.8–231)	0.17
Alb (g/dL)		4.2 (3.9–4.4)	4.1 (3.9–4.4)	4.2 (3.9–4.5)	0.22
Cre (mg/dL)		0.86 (0.71–1.00)	0.90 (0.76–1.00)	0.81 (0.71–0.99)	0.20
Na (mEq/L)		139 (138–140)	140 (137–141)	139 (138–140)	0.15
K (mEq/L)		4.2 (4.0–4.5)	4.3 (4.0–4.6)	4.2 (4.0–4.5)	0.43
Cl (mEq/L)		104 (102–106)	105 (103–106)	103 (102–105)	0.05
Ca (mg/dL)		9.1 (8.8–9.4)	9.1 (8.9–9.3)	9.1 (8.8–9.5)	0.59
CRP (mg/dL)		0.1 (0.0–0.2)	0.1 (0.0–0.1)	0.1 (0.0–0.2)	0.05
WBC (×10^3^/μL)		6010 (4840–6900)	5780 (4680–6640)	6150 (5000–6905)	0.29
Hb (g/dL)		13.6 (12.4–15.0)	13.2 (12.2–14.6)	13.8 (12.6–15.0)	0.22
Plt (×10^4^/μL)		20.4 (16.9–24)	19.3 (15.4–22)	21.5 (17.9–25.3)	0.007
Tumor size (mm)		15 (10–20)	20 (10–30)	15 (10–20)	0.006
Number of tumors					0.38
	Solitary	79 (56)	30 (38)	49 (62)	
	Multiple	62 (44)	19 (31)	43 (69)	
Tumor location					0.469
	Posterior wall	32 (23)	15 (47)	17 (53)	
	Lateral wall	73 (52)	22 (29)	51 (71)	
	Anterior wall	5 (3)	1 (20)	4 (80)	
	Trigone	10 (7)	5 (50)	5 (50)	
	Dome	6 (4)	2 (33)	4 (67)	
	Bladder neck	15 (10)	4 (27)	11 (73)	
Operation time (min)		50 (36–67)	55 (42–71)	40 (26–60)	< 0.001
Previous TURBT (*n*)					0.64
	1	97 (69)	31 (32)	66 (68)	
	2	31 (22)	13 (42)	18 (58)	
	≥3	13 (9)	5 (38)	8 (62)	
Complication					-
	I	28 (20)	28 (100)	0 (0)	
	II	13 (9)	13 (100)	0 (0)	
	≥III	8 (6)	8 (100)	0 (0)	
	0	92 (65)	92 (100)	0 (0)	
Pathological T stage					<0.001
	Ta	85 (60)	20 (23)	65 (77)	
	T1	23 (17)	11 (48)	12 (52)	
	Tis	7 (5)	4 (57)	3 (43)	
	≥T2	9 (6)	8 (89)	1 (11)	
	Others	17 (12)	11 (65)	6 (35)	
Cytology					0.005
	≤Class II	75 (53)	19 (25)	56 (75)	
	Class III	37 (26)	13 (35)	24 (65)	
	Class IV	12 (9)	7 (58)	5 (42)	
	Class V	16 (10)	10 (63)	6 (37)	
	No data	1 (1)	0 (0)	1 (100)	

^1^ Data are presented as median (IQR) or number (%). CCI, Charlson Comorbidity Index; ECOG PS, Eastern Cooperative Oncology Group performance status; BMI, body mass index; LDH, lactate dehydrogenase; ALP, alkaline phosphatase; Alb, albumin; Cre, creatinine; Na, sodium; K, potassium; Cl, chloride; Ca, calcium; CRP, C-reactive protein; WBC, white blood cell; Hb, hemoglobin; Plt, platelet count; TURBT, transurethral resection of bladder tumor.

**Table 2:** Postoperative complications according to the Clavien–Dindo classification.

Complications	Clavien–Dindo I	Clavien–Dindo ≥ II
Hematuria	19	15
Urinary retention	3	1
Bladder perforation	1	2
Urinary tract infection	1	1
Ureteral obstruction	1	1
Dysuria	2	0
Nausea	1	1
Total	28	21

**Table 3:** Univariate and multivariate logistic regression analyses of factors associated with postoperative complications.

Variables		Univariate Analysis			Multivariate Analysis		
		OR	95%CI	*p*	OR	95%CI	*p*
Age (years)		1.04	1.00–1.08	0.03	1.03	0.97–1.10	0.32
Sex	Female	Ref.	Ref.	Ref.			
	Male	0.95	0.40–2.25	0.91			
Alb (g/dL)		1.79	0.78–4.10	0.17			
ALP (U/L)		0.99	0.99–1.00	0.09			
BMI (kg/m^2^)		1.00	0.91–1.10	0.97			
Na (mEq/L)		1.06	9.26 × 10^–0.1^–1.21	0.42			
K (mEq/L)		1.65	0.63–4.33	0.31			
Ca (mg/dL)		0.84	0.38–1.91	0.69			
Cl (mEq/L)		1.07	0.95–1.21	0.26			
Cre (mg/dL)		1.67	0.66–4.23	0.28			
CRP (mg/dL)		0.67	0.31–1.44	0.31			
WBC (×10^3^/μL)		1.00	1.00–1.00	0.20			
Hb (g/dL)		0.89	0.74–1.07	0.20			
Plt (×10^4^/μL)		0.91	0.85–0.98	<0.01	0.89	0.82–0.96	<0.01
ECOG PS	0	Ref.	Ref.	Ref.			
	≥1	2.37	0.80–6.98	0.12			
Cytology	≤Class II	Ref.	Ref.	Ref.			
	≥Class III	2.69	1.32–5.50	<0.01	1.72	0.76–3.89	0.19
Tumor location	Posterior wall	Ref.	Ref.	Ref.			
	Lateral wall	0.49	0.21–1.15	0.10			
	Anterior wall	0.57	0.10–3.55	0.54			
	Trigone	1.13	0.27–4.69	0.86			
	Dome	0.28	0.02–2.82	0.28			
	Bladder neck	0.41	0.11–1.57	0.19			
Tumor number	Solitary	Ref.	Ref.	Ref.			
	Multiple	1.39	0.68–2.81	0.37	2.68	0.76–6.61	0.32
Pathological T stage	≤T1	Ref.	Ref.	Ref.			
	≥T2	5.79	1.46–23	0.01	5.51	1.23–25	<0.01
CCI		1.11	0.92–1.32	0.27			
Tumor size (mm)		1.05	1.01–1.08	<0.01	1.05	1.02–1.09	<0.01
Previous TURBT (*n*)		1.22	0.90–1.66	0.21	1.44	1.02–2.03	0.40
Operation time (min)		1.02	1.01–1.04	<0.01	1.01	1.00–1.02	0.14

OR, odds ratio; CI, confidence interval; Alb, albumin; ALP, alkaline phosphatase; BMI, body mass index; Na, sodium; K, potassium; Ca, calcium; Cl, Chloride; Cre, creatinine; CRP, C-reactive protein; WBC, white blood cell; Hb, hemoglobin; Plt, platelet; ECOG PS, Eastern Cooperative Oncology Group performance status; CCI, Charlson Comorbidity Index; TURBT, transurethral resection of bladder tumor.

## Data Availability

The data presented in this study is available on request from the corresponding author.

## Abbreviations

The following abbreviations are used in this manuscript:

ALP	alkaline phosphatase
BMI	body mass index
CI	confidence interval
CCI	Charlson comorbidity index
CIS	carcinoma in situ
CRP	C-reactive protein
OR	odds ratio
TUR	transurethral resection
TURBT	transurethral resection of bladder tumor
WBC	white blood cell
